# Supplementary material for: Generalized Linear Models outperform commonly used canonical analysis in estimating spatial structure of presence/absence data
Source: PeerJ. 2020 Sep 3;8:e9777. doi: 10.7717/peerj.9777 (PMC7474884; doi:10.7717/peerj.9777)
Supplement: Supplemental Information 7 [file peerj-08-9777-s007.pdf]

Supporting Article S1.

Real spatial structure in simulated data vs. structures selected by GLM/AIC and RDA/FW

The main utility of generating eigenvector-based spatial variables is to assess linear combinations of variables that can uncover the spatial scale(s) on which biological communities are structured. One usual approach to explore these spatial structures is using maps to plot the MEM variable decompositions. Below, for each dataset (A = subtidal algae from Ilha Grande Bay; B= Scotland grasslands; C= riverine macroinvertebrates) we have explored the differences between the spatial structure simulated within the ecological data and the structures selected by both methods being tested, GLM/AIC and RDA/FW.

Each dataset has been depicted by a graph showing connectivity between sites, modelled as described in the methods. For dataset A, the chosen connectivity graph was the minimum spanning tree; the Delaunay triangulation was used for dataset B and asymmetric eigenvector maps were used to describe the spatial relationships among the sampling sites in dataset C (Fig. S1.1). We have picked for each dataset those conditions in which each method (GLM/AIC and RDA/FW) performed worst (Table S1.1). These were extracted from results shown in Figure 4 (see main text) and a table describing each of these conditions for all datasets is provided below. Finally, we plotted the true pattern associated with each spatial explanatory variable by its MEM decomposition, along with the corresponding estimated patterns from one haphazardly chosen replicate simulation. The plot consists of maps showing squares of different sizes that are proportional to the scores for each sampling site associated to that MEM variable. Open squares represent negative and filled squares represent positive values.

In MEMs, larger eigenvalues are associated with broader-scale spatial structures while smaller eigenvalues represent fine-scale spatial structures, and they are ranked in descending order of spatial scale (*i.e.* MEM 1 represents the broadest spatial structure, MEM 2 the second broadest and so on). Finally, in each plot the eigenvalue associated with each MEM variable is shown. Including variables associated with substantially different eigenvalues could lead to wrong interpretation of the scales on which these communities are structured.

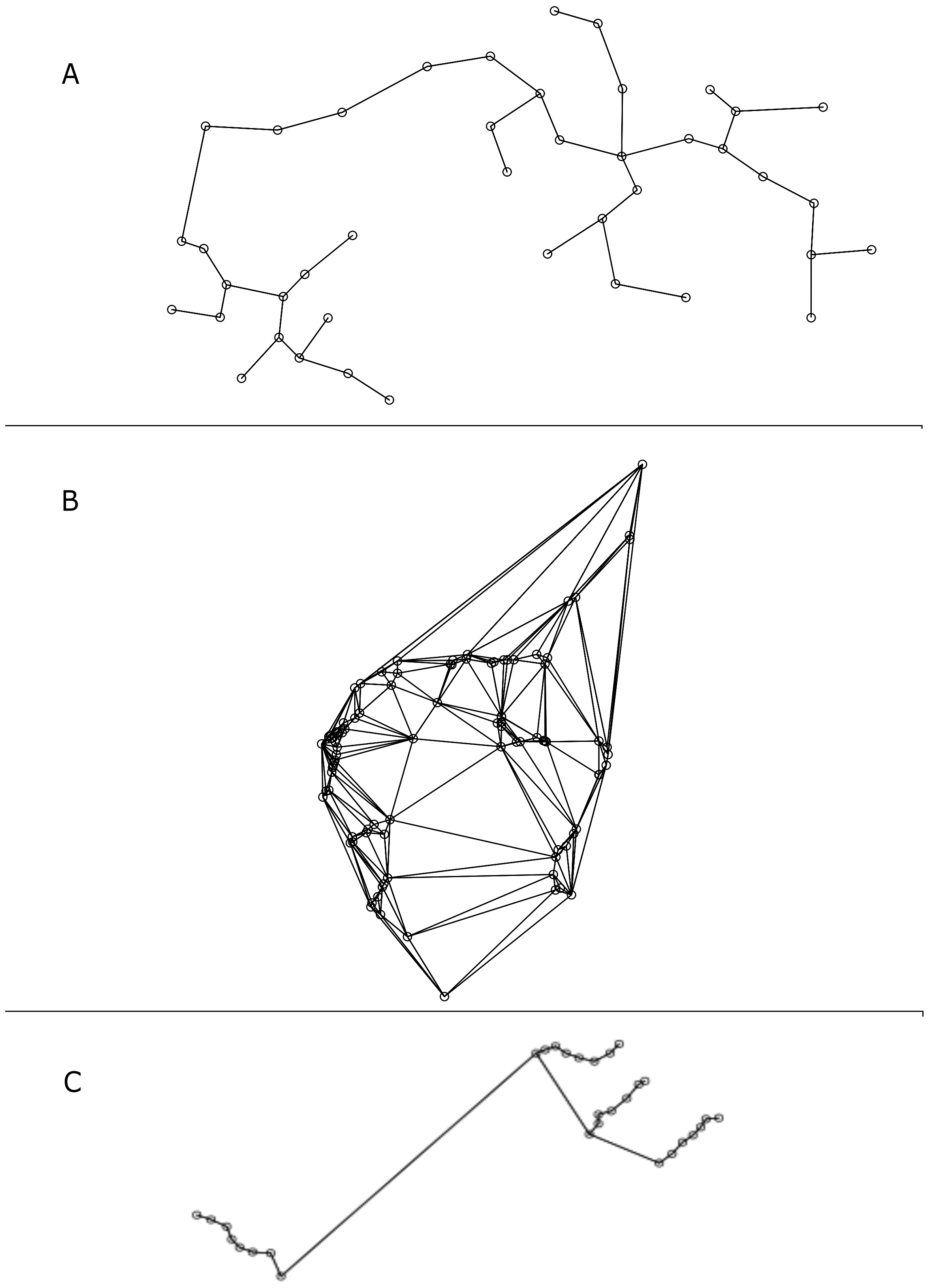

Figure S1. 1 Spatial distribution and connectivity of sampling sites for the three baseline datasets: subtidal algae community (A), Scotland grasslands (B) and freshwater insects (C). The sites were connected by different methods, according to the intrinsic ecological relationship among sites: minimum spanning tree in dataset A, Delaunay triangulation in dataset B and a vector describing the water flow of the river used for asymmetric eigenvector maps in dataset C.

Table S1.1. Selected models (based on the worst performance of each method for all datasets, as depicted in Figure 4 from main text

	Worst-performing modelling scenario	
Dataset:	GLM/AIC	RDA/FW
A) Algae	nVar=16 (scaling 1)	nVar=5 (scaling 2)
B) Scotland grassland	nVar = 30 (scaling 1)	nVar=10 (scaling 1)
C) Freshwater insects	nVar = 12 (scaling 1)	nVar = 2 (scaling 1)

1. **Real spatial structure in simulated data vs. structures selected by GLM/AIC.**

For the algae dataset (A), GLM/AIC performed worst in the simulation scenario in which it should have selected all 16 MEM variables (Table S1.1; Fig S1.2), *i.e.* where the simulated community was structured at all (broad, intermediate and fine) spatial scales. Under this simulation scenario, GLM/AIC only selected 11 variables (Fig. S1.3). However, the spatial structure retrieved by GLM/AIC contained variables representing patterns at broad (e.g. MEMs 2 and 4), intermediate (e.g. MEMs 8 and 9) and fine scales (e.g. MEMs 14-16). Therefore, the general spatial structure was captured by the model selection process.

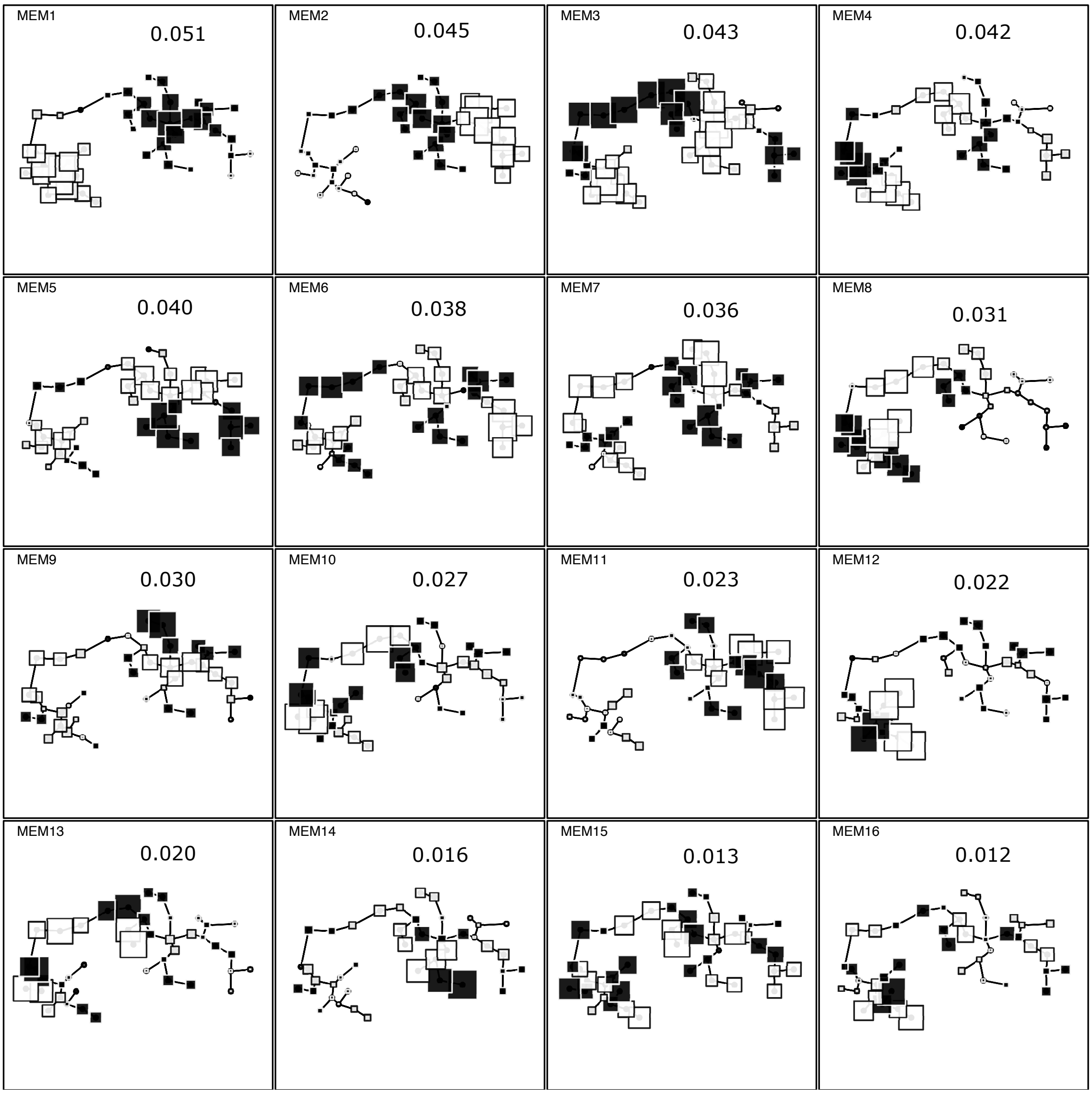

***Figure S1. 2** Spatial pattern in simulated subtidal algae data decomposed into MEMs, nvar = 16. The eigenvalues associated with each MEM variable are represented on the upper right side of each panel. All values were rounded to three decimal places.*

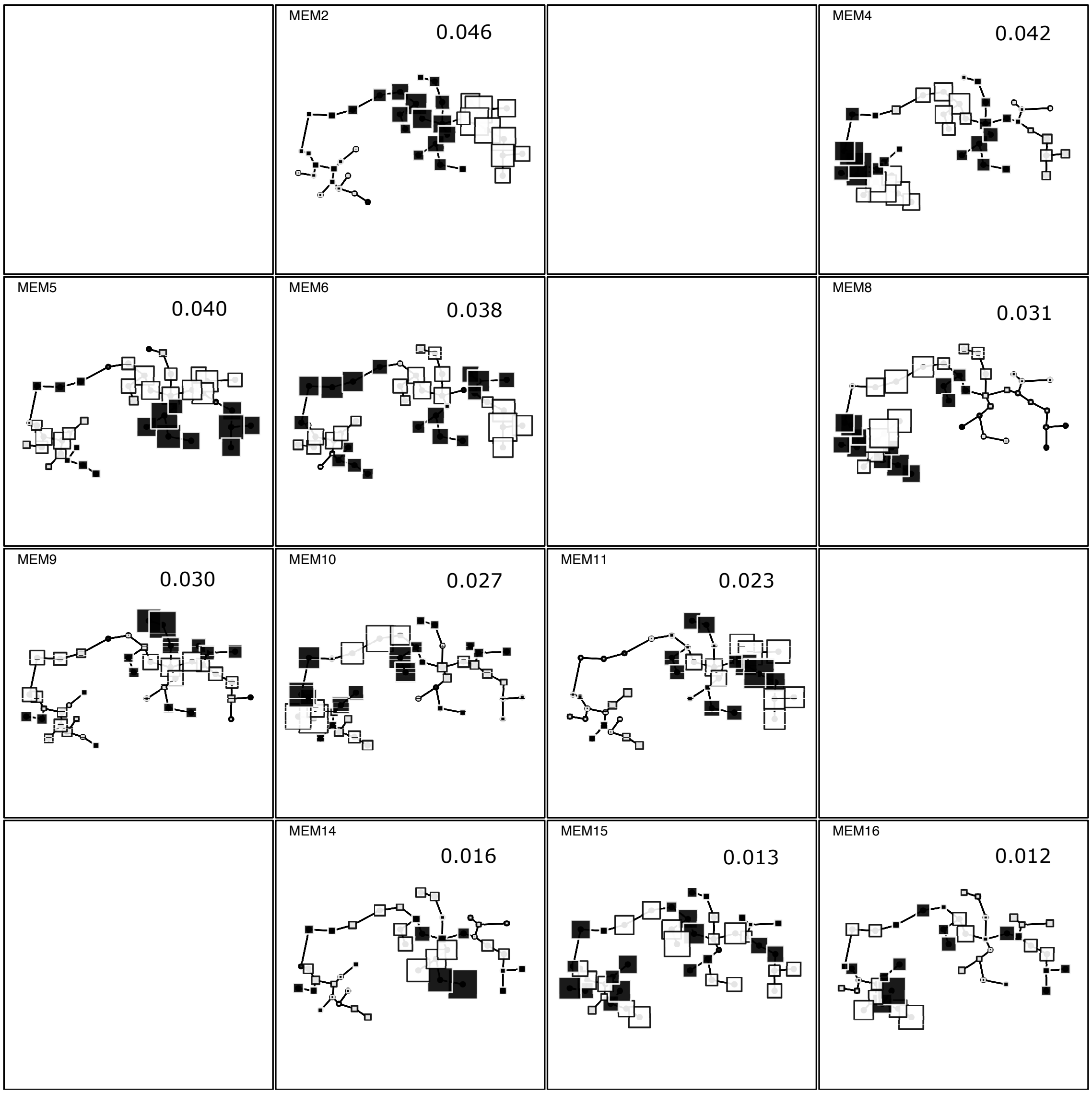

Figure S1. 3 *Spatial structures selected by GLM/AIC decomposed into MEMs. The eigenvalues associated with each MEM variable are represented on the upper right side of each panel. All values were rounded to three decimal places.*

Similarly, for the Scotland grassland dataset (B), GLM/AIC performed worst when it was supposed to select all 30 MEMs (Fig. S1.4), but instead retrieved only 23 MEMs (Fig. S1.5). Again, the selected variables encompassed broad, intermediate and fine scaled spatial patterns.

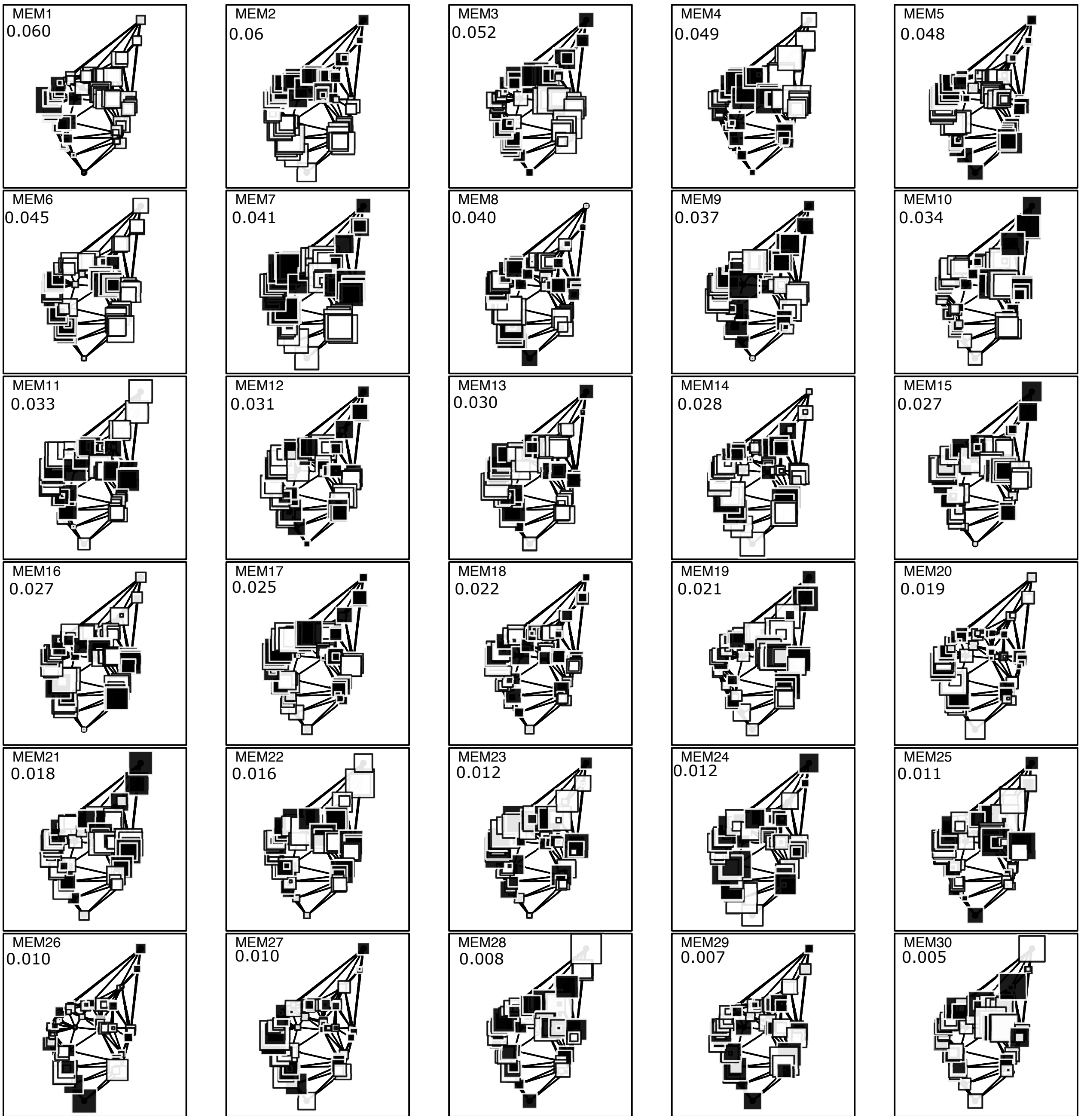

Figure S1. 4 *Spatial pattern in simulated Scotland grassland data decomposed into MEMs, nvar = 30. The eigenvalues associated with each MEM variable are represented on the upper left side of each panel. All values were rounded to three decimal places.*

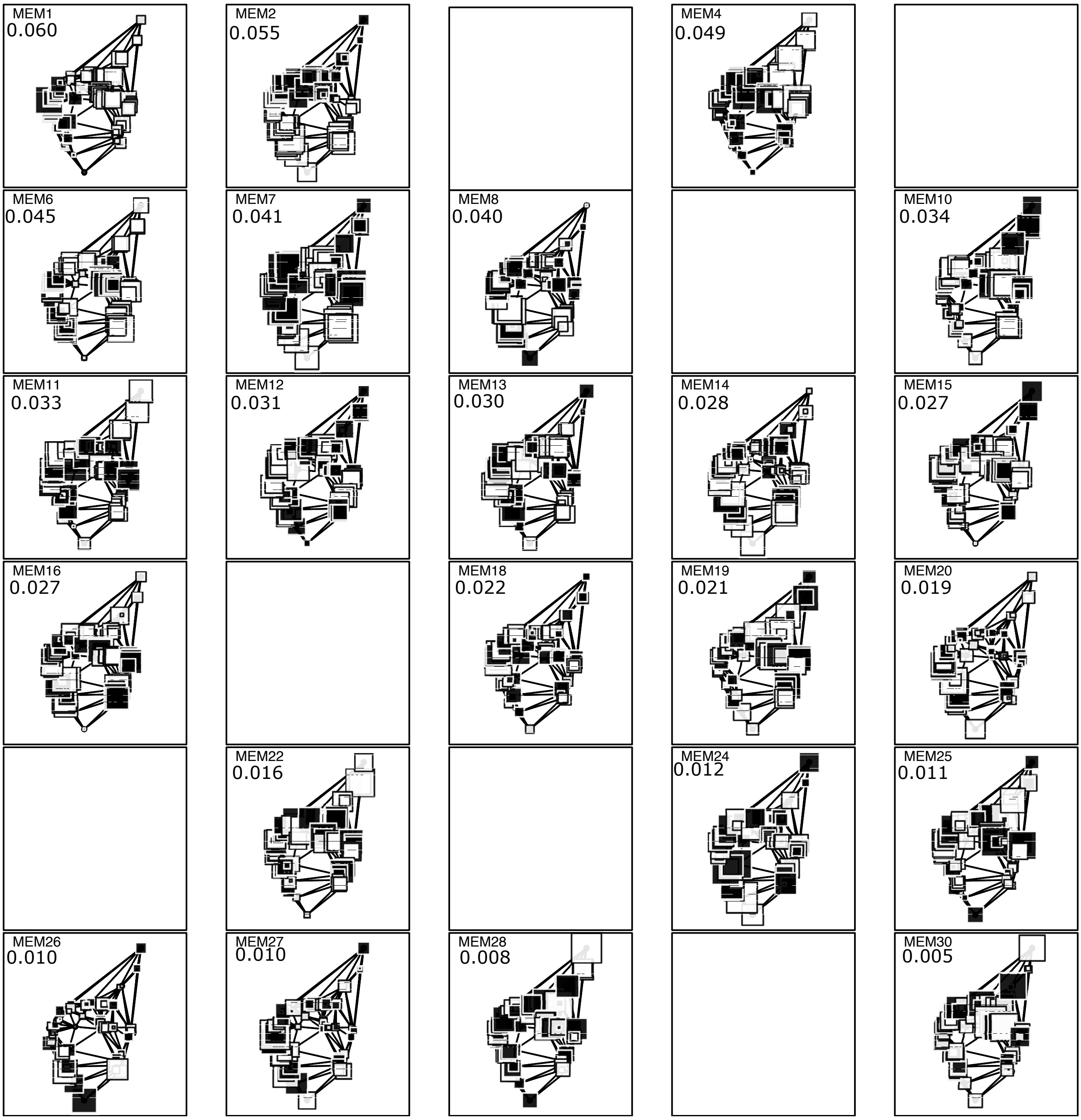

Figure S1. 5 *Spatial structures selected by GLM/AIC decomposed into MEMs. The eigenvalues associated with each MEM variable are represented on the upper left side of each panel. All values were rounded to three decimal places.*

For the freshwater insects dataset (C), GLM/AIC performed worst when it was supposed to select all 12 MEMs (Fig. S1.6), but instead retrieved only seven MEMs (Fig. S1.7). Again, the selected variables encompassed broad, intermediate and fine scaled spatial patterns.

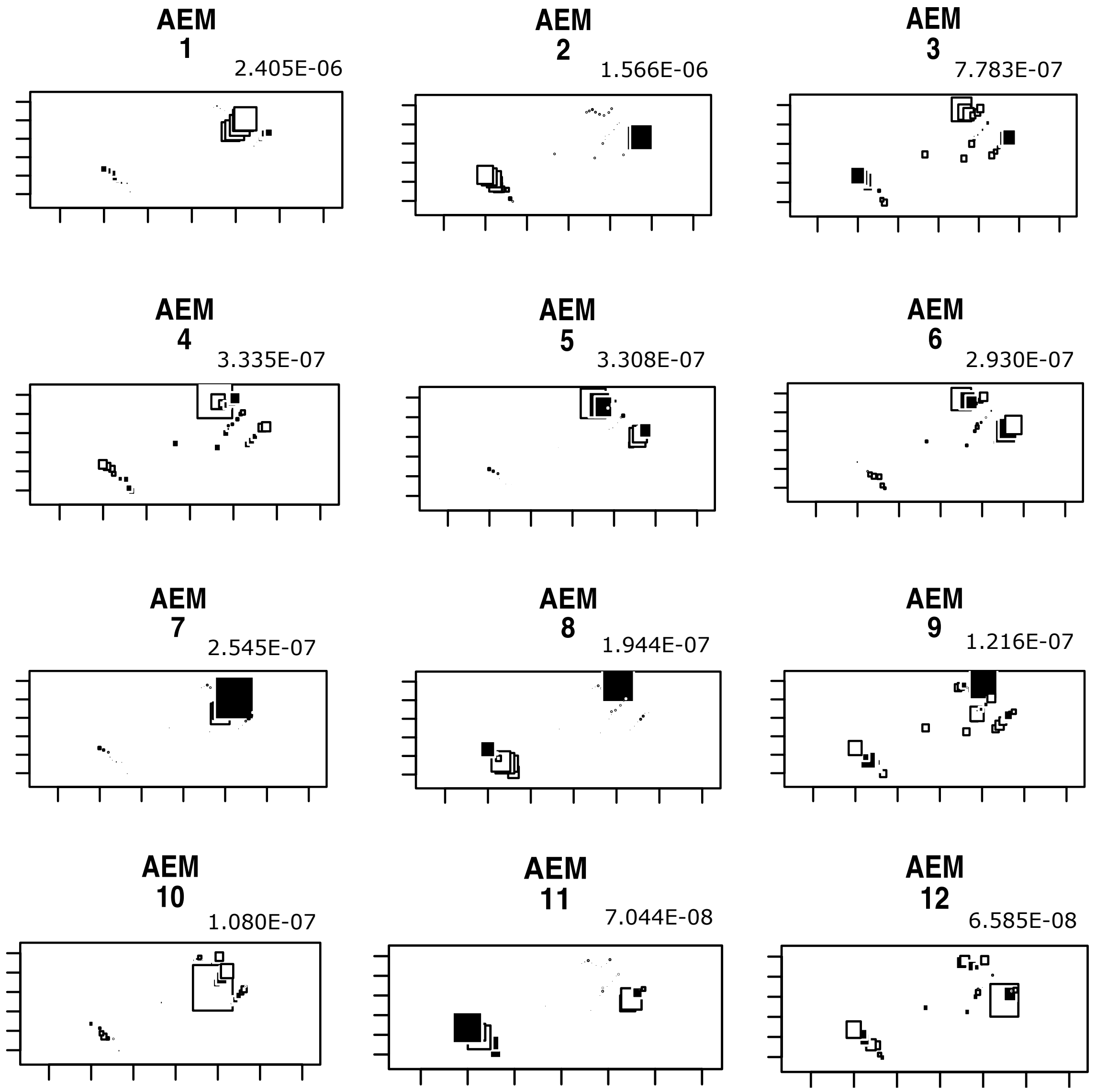

Figure S1. 6 *Spatial pattern in simulated freshwater insect data decomposed into MEMs, nvar = 12. The eigenvalues associated with each MEM variable are represented on the upper right side of each panel. All values were rounded to three decimal places.*

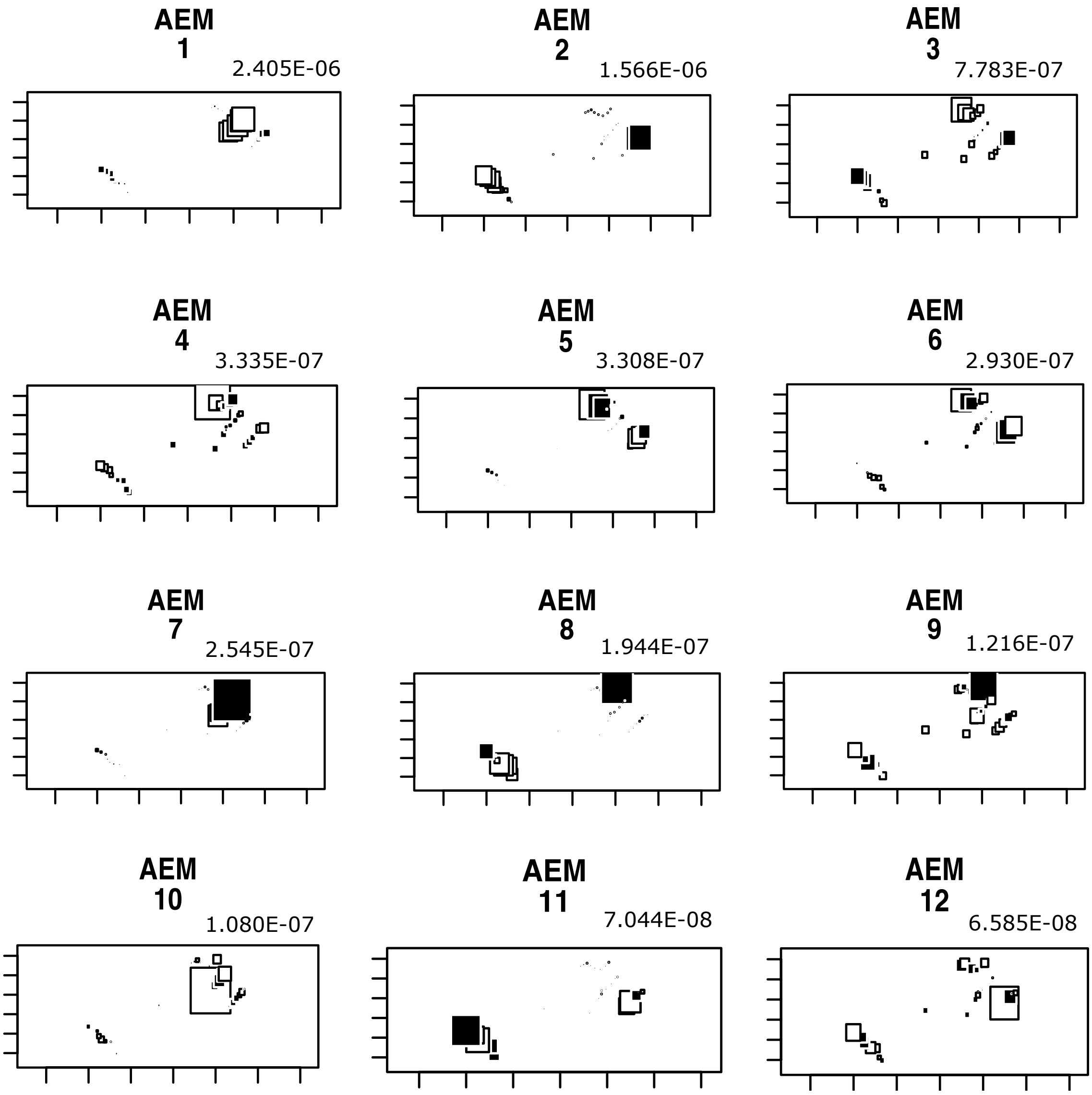

Figure S1. *7 Spatial structures selected by GLM/AIC decomposed into MEMs. The eigenvalues associated with each MEM variable are represented on the upper right side of each panel. All values were rounded to three decimal places.*

2. Real spatial structure in simulated data vs. structures selected by RDA/FW.

Contrary to GLM/AIC, RDA/FW typically included more variables than it should. For example, in the algae dataset, when the simulated community was structured by the last, fine-scaled, five MEMs (MEMs 12,13,14,15,16; Fig. S1.8), RDA/FW wrongly selected 13 MEMs (MEMs 1, 2, 3, 4, 5, 6, 9, 11, 12, 13, 14, 15, 16, Fig. S1.9). The variables incorrectly included were associated with the broadest scales among the MEM variables (MEMs 1, 2 and 3). Mistaking fine for broad-scale structures (or vice-versa) would make it difficult to reveal the spatial patterns present in the response data and lead to misinformed discussions and conclusions about the spatial scales on which communities are organized.

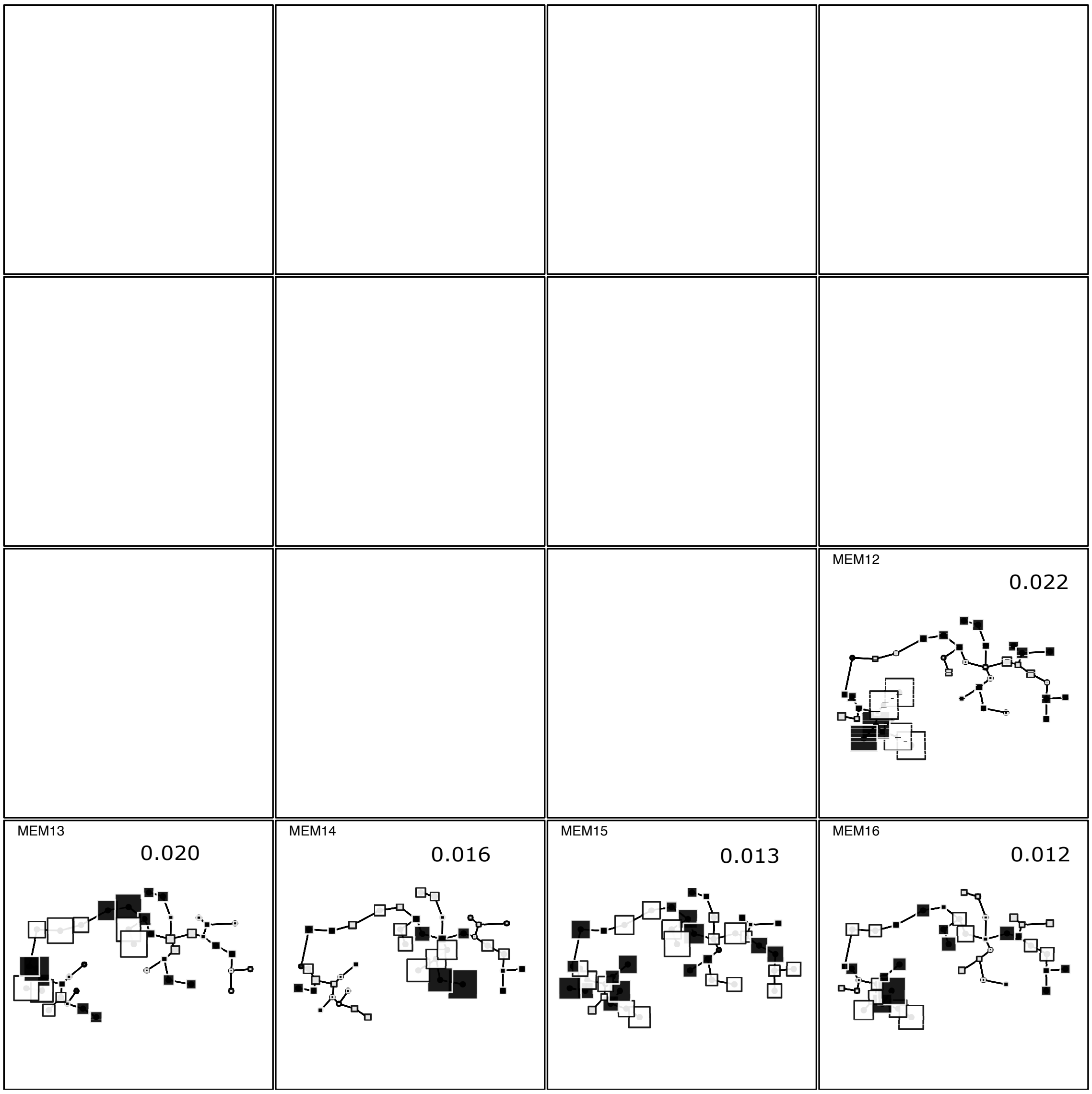

Figure S1. 8 *Spatial pattern in simulated freshwater insect data decomposed into MEMs, nvar = 5 in scaling 2 (i.e. containing only fine scale variables, see main text). The eigenvalues associated with each MEM variable are represented on the upper right side of each panel. All values were rounded to three decimal places.*

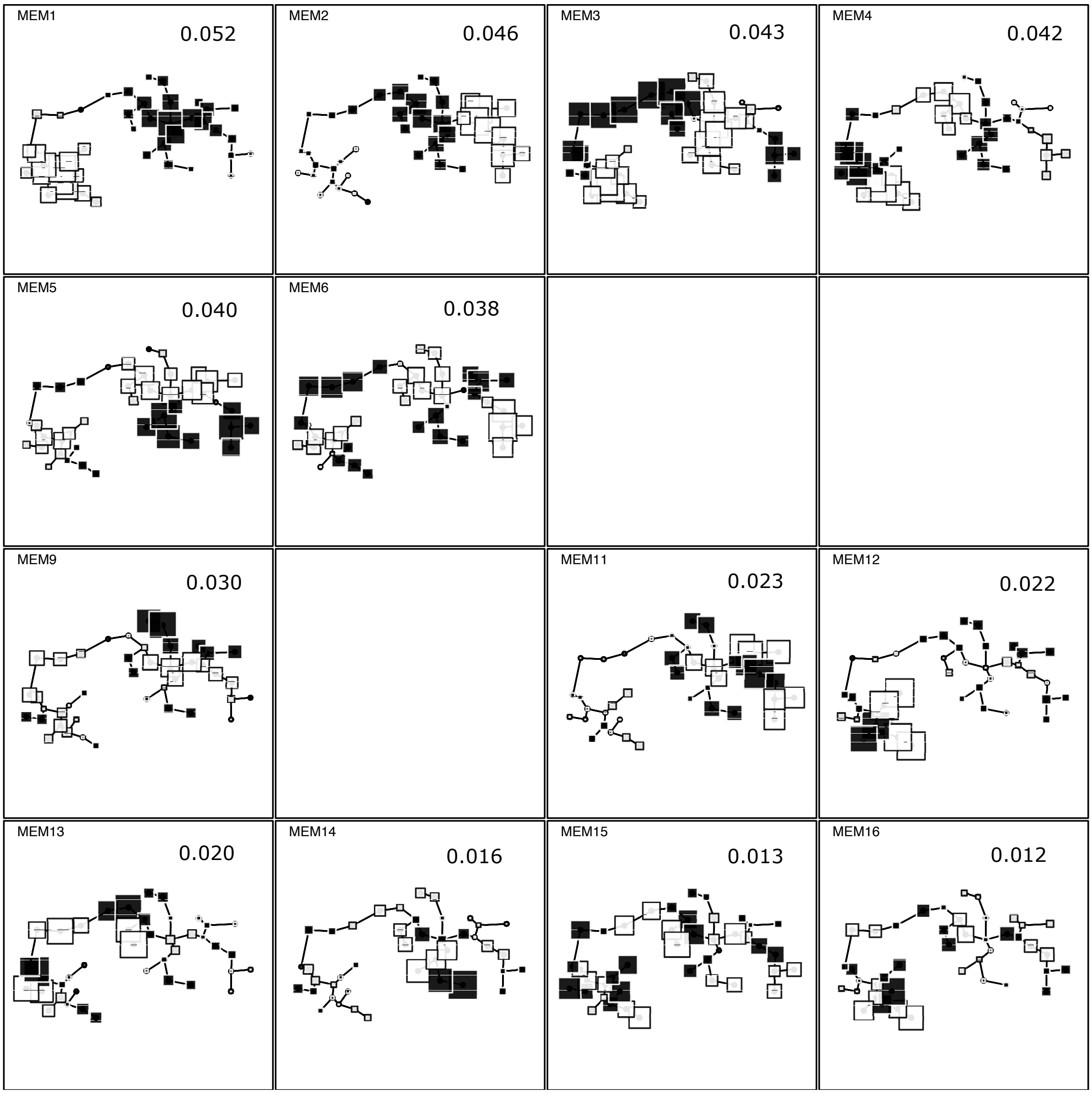

Figure S1. 9 *Spatial structures selected by RDA/FW decomposed into MEMs. The eigenvalues associated with each MEM variable are represented on the upper right side of each panel. All values were rounded to three decimal places.*

The same occurred for the Scotland grassland dataset. When the simulated community was structured by the first 10 MEMs, (MEMs 1-10; Fig. S1.10), RDA/FW wrongly selected the first 28 MEMs, leaving only two non-zero coefficient variables out of the final model (MEMs 1-28, Fig. S1.11). Although the simulated data were only structured on broad some intermediate spatial scales, RDA/FW wrongly included several fine-scale spatial patterns (e.g. MEMs 20-28).

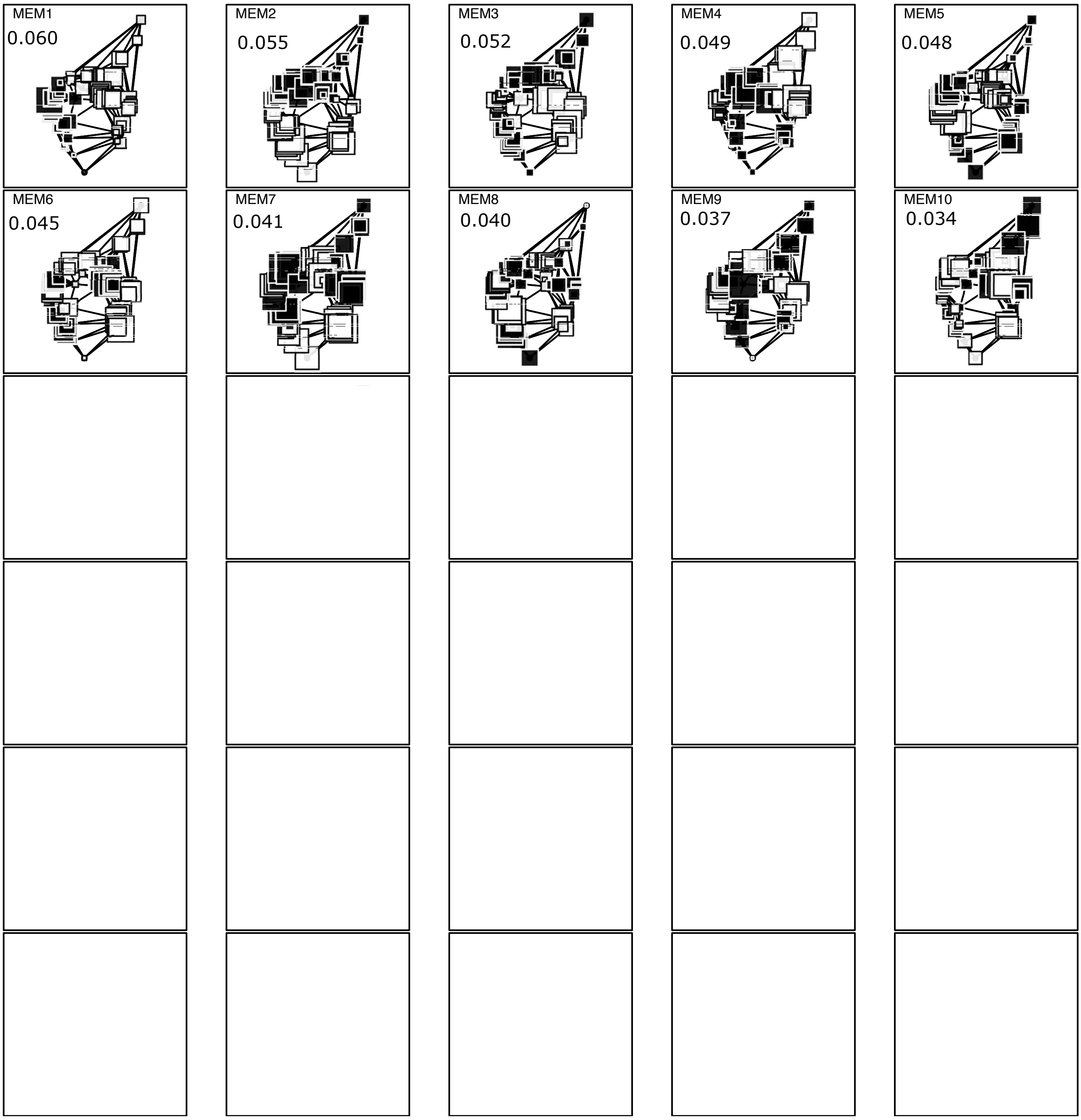

Figure S1. 10 *Spatial pattern in simulated Scotland grasslands data decomposed into MEMs, nvar = 10 in scaling 1 (i.e. containing only broad scale variables, see main text). The eigenvalues associated with each MEM variable are represented on the upper left side of each panel. All values were rounded to three decimal places.*

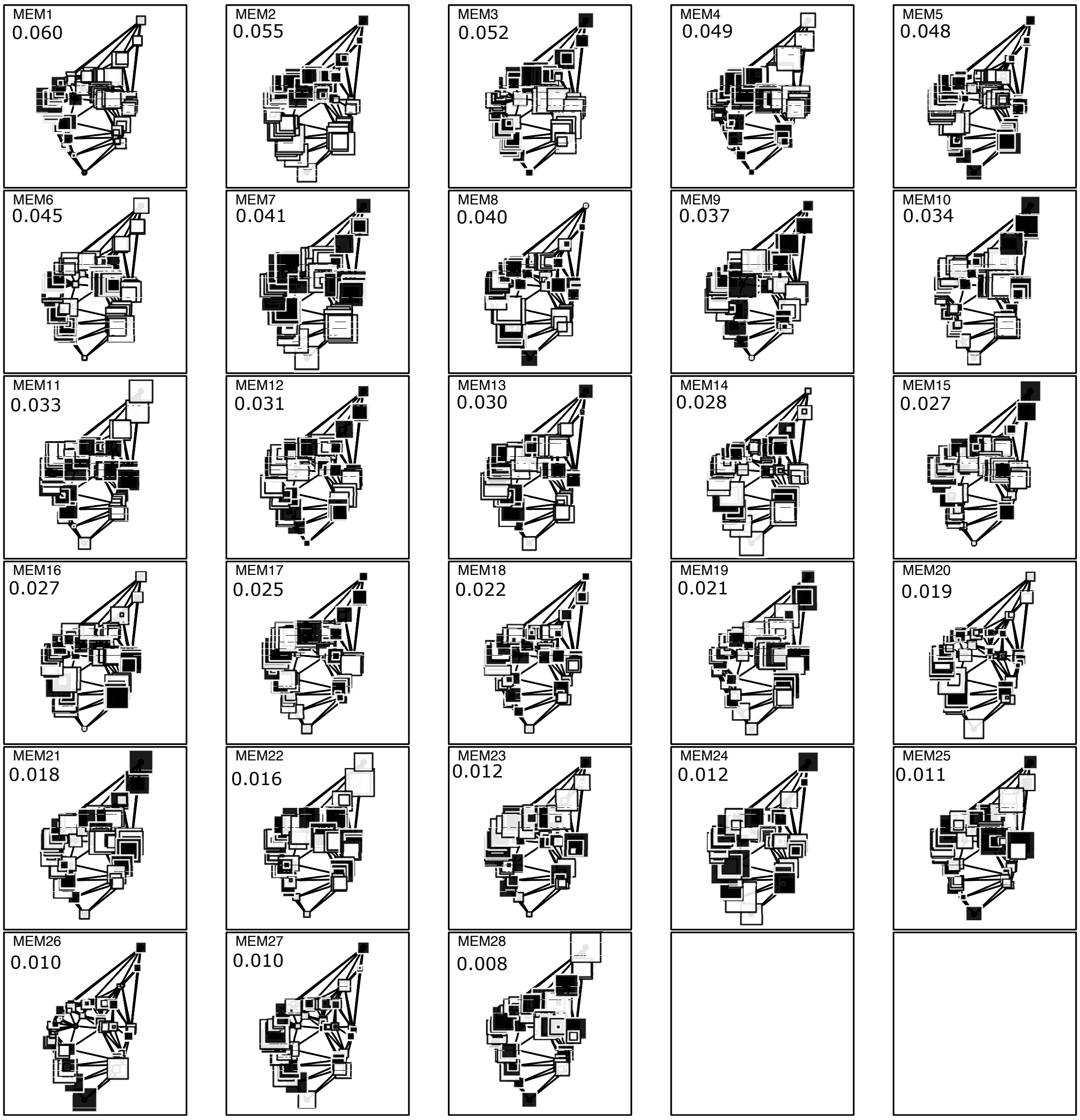

Figure S1. 11 *Spatial structures selected by RDA/FW decomposed into MEMs. The eigenvalues associated with each MEM variable are represented on the upper left side of each panel. All values were rounded to three decimal places.*

In dataset C, again RDA/FW consistently included variables that should not have been incorporated to the final model. When the freshwater insect community data was structure by only the two broadest AEM variables (Fig. S1.12), RDA/FW selected six variables (AEMs 1, 2, 3, 6, 8 and 10; Fig. S1.13), also incorporating intermediate (e.g. AEM 6) and fine-scaled patterns (e.g. AEM 10).

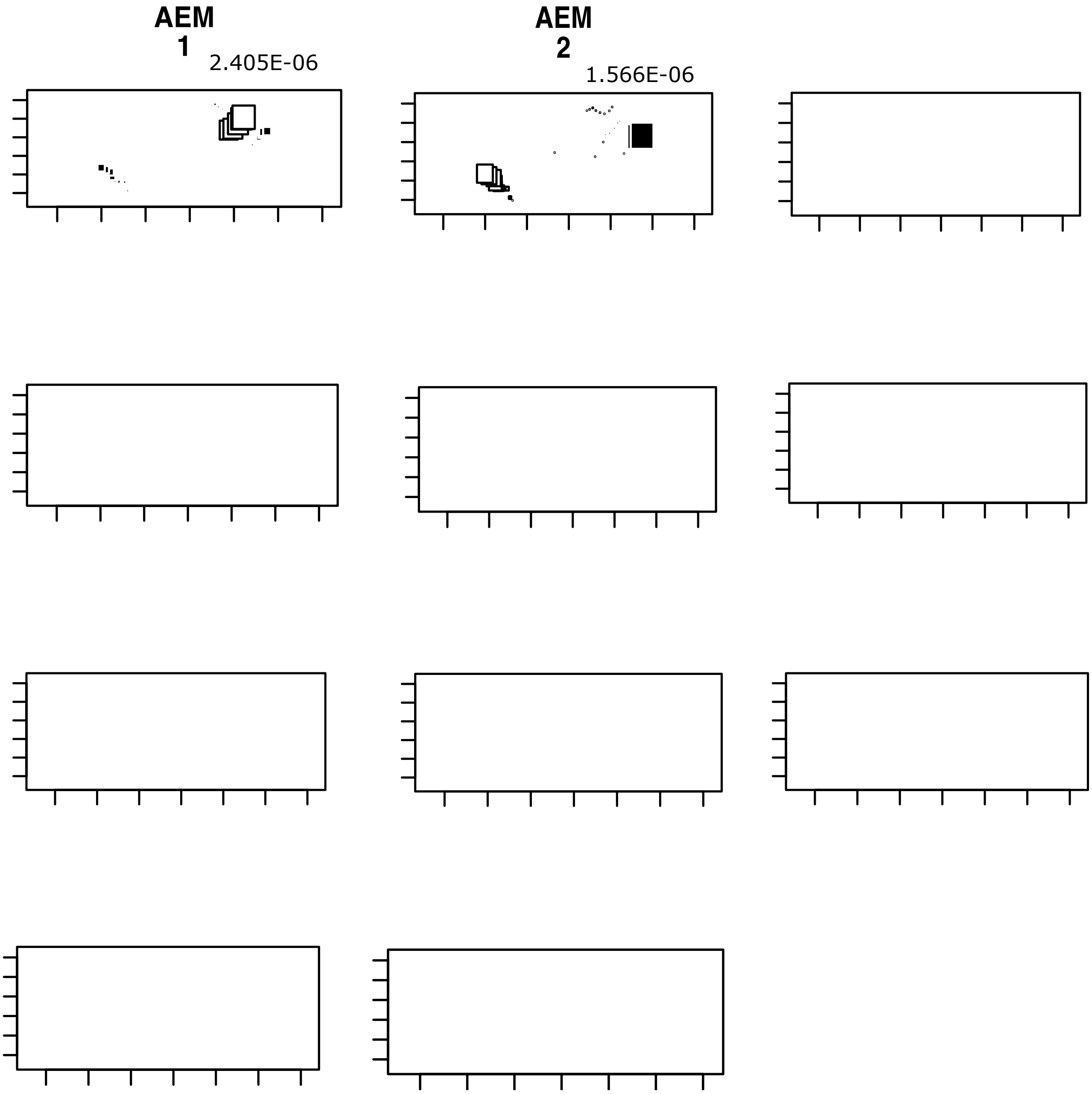

__Figure S1. 12__ Spatial pattern in simulated freshwater insect data decomposed into AEMs, nvar = 2 in scaling 1 (i.e. containing only broad scale variables, see main text). The eigenvalues associated with each MEM variable are represented on the upper right side of each panel. All values were rounded to three decimal places.

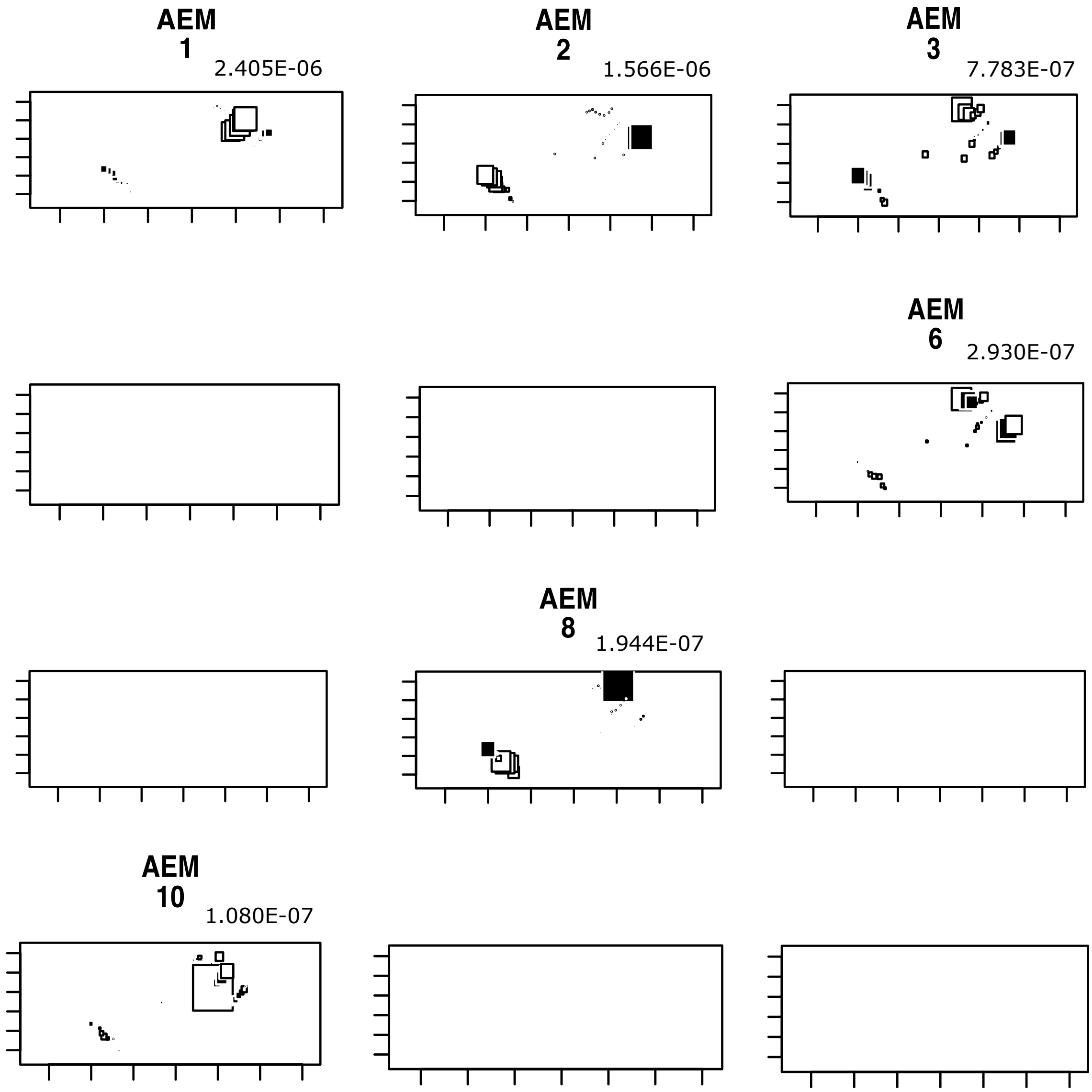

Figure S1. 13 *Spatial structures selected by RDA/FW decomposed into AEMs. The eigenvalues associated with each MEM variable are represented on the upper left side of each panel. All values were rounded to three decimal places.*

Plotting eigenvalues in rank order gives a little more information on the scales of spatial pattern. In the algae and Scotland data (Fig. S1. 14 A and B, respectively), eigenvalues decreased steadily in rank order, so that differences in rank order (as examined in the MEM decompositions above) are a reliable guide to differences in spatial scale. In the freshwater insect data (Fig. S1. 14 C), there were larger differences among the first three eigenvalues than among the last nine, but incorrectly selecting variables from among the last nine when only the first two should be present (as in Fig. S1. 13, for RDA/FW) gives a very misleading picture of spatial scale. In the algae data the ratio of largest to smallest eigenvalues was relatively small (4.3), so the inclusion of a few extra variables would presumably not be very detrimental to general conclusions about spatial patterns. However, the ratio of largest to smallest eigenvalues was larger for the Scotland data (11.7) and especially the freshwater insect data (36.5), so that selecting the wrong variables would make more difference to conclusions about the scale of spatial pattern.

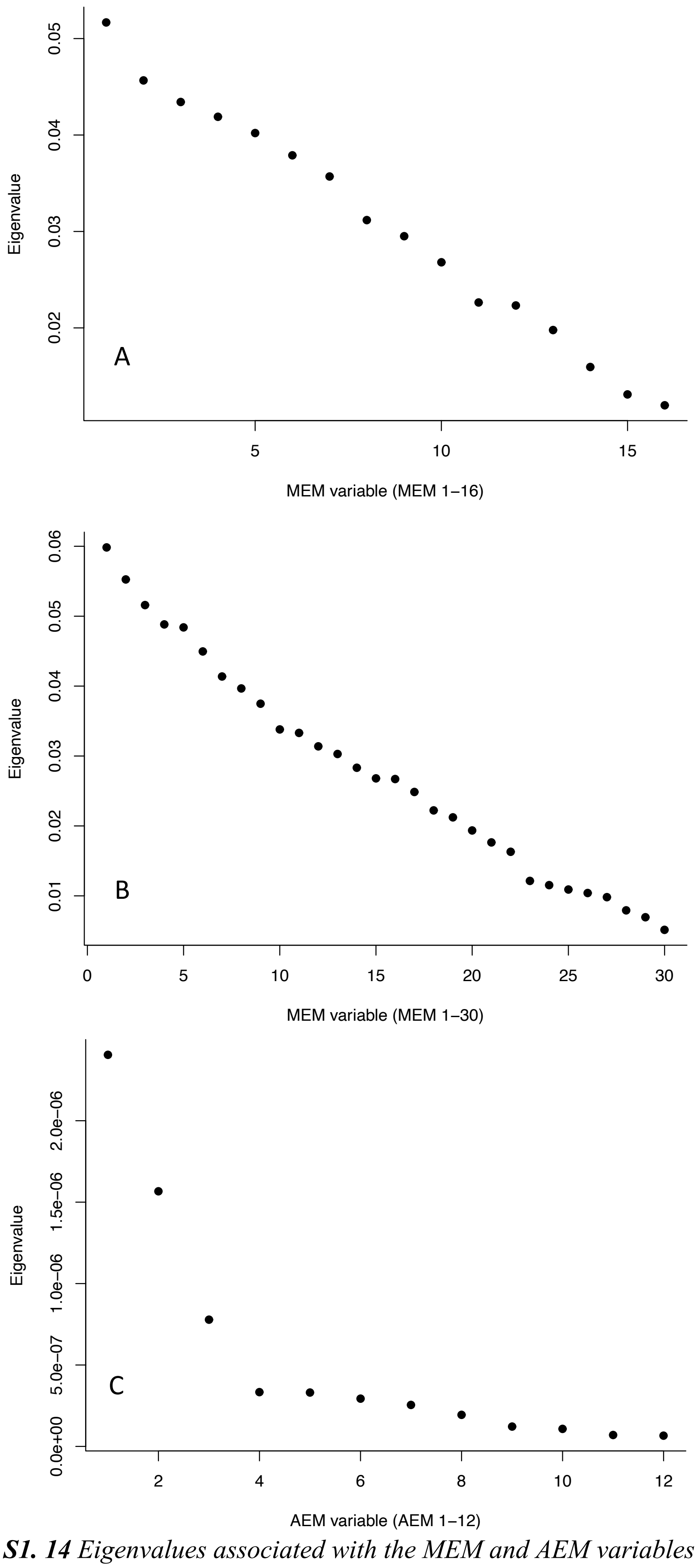

Figure S1. 14 *Eigenvalues associated with the MEM and AEM variables of the macroalgae (A = 16 eigenvalues), Scotland grasslands (B = 30 eigenvalues) and freshwater insects (C = 12 eigenvalues) datasets.*
